# Racial and ethnic differences in personal cervical cancer screening amongst post-graduate physicians: Results from a cross-sectional survey

**DOI:** 10.1186/1471-2458-8-378

**Published:** 2008-10-30

**Authors:** Joseph S Ross, Marcella Nuñez-Smith, Beverly A Forsyth, Julie R Rosenbaum

**Affiliations:** 1 Department of Geriatrics and Adult Development, Mount Sinai School of Medicine, and HSR&D Targeted Research Enhancement Program and Geriatrics Research, Education, and Clinical Center, James J. Peters Veterans Administration Medical Center, Bronx, NY, USA; 2 Robert Wood Johnson Clinical Scholars Program, Department of Internal Medicine, Yale University School of Medicine, New Haven, CT, USA; 3 Division of Infectious Diseases, Department of Medicine, Mount Sinai School of Medicine, NY, USA; 4 Primary Care Internal Medicine Residency Program, Yale University School of Medicine, and Department of Medicine, Waterbury Hospital Health Center, New Haven, CT, USA

## Abstract

**Background:**

Racial and ethnic disparities in cervical cancer screening have been attributed to socioeconomic, insurance, and cultural differences. Our objective was to explore racial and ethnic differences in adherence to cervical cancer screening recommendations among female post-graduate physicians.

**Methods:**

We conducted a cross-sectional survey at one university hospital among a convenience sample of 204 female post-graduate physicians (52% of all potential participants), examining adherence to United States Preventive Services Task Force cervical cancer screening recommendations, perception of adherence to recommendations, and barriers to obtaining care.

**Results:**

Overall, 83% of women were adherent to screening recommendations and 84% accurately perceived adherence or non-adherence. Women who self-identified as Asian were significantly less adherent when compared with women who self-identified as white (69% vs. 87%; Relative Risk [RR] = 0.79, 95% Confidence Interval [CI], 0.64–0.97; P < 0.01). Women who self-identified as East Indian were significantly less likely to accurately perceive adherence or non-adherence when compared to women who self-identified as white (64% vs. 88%; RR = 0.73, 95% CI, 0.49–1.09, P = 0.04). Women who self-identified as Asian were significantly more likely to report any barrier to obtaining care when compared with women who self-identified as white (60% vs. 35%; RR = 1.75, 95% CI, 1.24–2.47; P = 0.001) and there was a non-significant tendency toward women who self-identified as East Indian being more likely to report any barrier to obtaining care when compared with women who self-identified as white (60% vs. 34%; RR = 1.74, 95% CI, 1.06–2.83; P = 0.06).

**Conclusion:**

Among a small group of insured, highly-educated physicians who have access to health care, we found racial and ethnic differences in adherence to cervical cancer screening recommendations, suggesting that culture may play a role in cervical cancer screening.

## Background

Racial and ethnic disparities in cancer diagnosis and prognosis have been well documented.[1] One explanation is differing utilization of preventive services. African-Americans are less likely than whites to receive breast cancer screening,[2] while Asians are less likely than whites, African-Americans or Latinas to receive breast[3] or cervical cancer screening.[4] In addition, Latinas are less likely than white non-Latinas to receive cervical cancer screening.[5]

Many factors have been associated with decreased cervical cancer screening, including obesity[6,7] and older age,[8] despite there being broad, general agreement as to the importance and effectiveness of screening for cervical cancer. [9-11] Specific factors such as low socioeconomic status, lack of insurance, and lack of a usual source of care have been associated with decreased cervical cancer screening among minorities and may contribute to racial and ethnic disparities, [12-14] as might cultural differences [15-17] or acculturation.[18,19]

The challenge of disentangling racial and ethnic differences in cervical cancer screening from differences related to socioeconomic status, education, insurance, or acculturation is immense. However, by studying cervical cancer screening among female post-graduate physicians, we had a unique opportunity to examine a group of insured, highly-educated women who have access to care and professional experience in cervical cancer and prevention. Physician preventive health care utilization has not been widely studied, but in instances when it has, some studies have found physicians to use preventive care services at high rates, [20-22] while others have found low rates of use.[23,24] To our knowledge, racial and ethnic differences in health care utilization among physicians have not been studied. The objective of our study was to explore adherence to cervical cancer screening recommendations among this racially and ethnically diverse population. Our hypothesis was that there would not be racial and ethnic differences in adherence to cervical cancer screening recommendations in this population of insured, highly-educated women who, as physicians, have health care expertise.

## Methods

A cross-sectional survey was administered to female post-graduate physicians at Montefiore Medical Center, the University Hospital of the Albert Einstein College of Medicine, Bronx, NY, identified using a register of all trainees. All female post-graduate physicians were eligible for participation. Between May 2003 and January 2004, women were approached in-person by an investigator (either JSR or BAF, who were post-graduate physicians at the institution at that time and knew some, but not all, of the women) to complete a self-administered, anonymous survey examining "health behaviors." Most housestaff were approached while alone in the hospital's medical library or cafeteria. After consenting to participate, housestaff were given an unmarked survey and an envelope that contained other completed surveys. The investigator stepped away for complete privacy and retrieved the envelope after the survey had been completed and placed with the others in the envelope, which required approximately five minutes. Completed surveys were not taken out of the envelope until the project's data collection phase was finished in order to further protect participants' privacy. Surveys were not distributed by electronic or inter-office mail because of response rate concerns. Institutional review board approval was obtained prior to the study.

The survey assessed socio-demographic characteristics, including self-identified race/ethnicity, and had been piloted for readability but not validated. Multiple-choice questions were used to assess the following primary outcomes: 1) adherence to United States Preventive Services Task Force (USPSTF) recommendations for cervical cancer screening, 2) perception of personal adherence to recommendations, and 3) barriers to obtaining care among women who perceived themselves to be non-adherent to USPSTF recommendations. The following barrier responses were provided: no health insurance; no physician; not comfortable having Pap smear at own institution; no time; not sexually active; test inaccurate; and other (with space provided). Where appropriate, respondents could select more than one proposed response and additional space was provided for open-ended responses.

The USPSTF gave cervical cancer screening an A recommendation, using indirect evidence to determine that screening women with a cervix within three years of sexual activity (or age 21) and at least every three years thereafter captures most of the benefit in terms of reducing morbidity and mortality.[11] However, USPSTF qualifies that women not receiving appropriate follow-up after an abnormal Pap smear are most at risk. For these reasons, with the additional understanding that both the American Cancer Society (ACS) and the American College of Obstetrics and Gynecology (ACOG) recommend annual screening,[9,10] we interpreted the USPSTF guidelines to suggest annual screening for women with a history an abnormal Pap smear. Therefore, when determining current adherence to cervical cancer screening recommendations, women who were sexually active but had never had a Pap smear and women with a history of an abnormal Pap smear were categorized as requiring screening in the past year. All others were categorized as requiring screening at least once in the past three years. We did not categorize women who were screened more than once within the past three years as non-adherent.

Data analysis was performed using SPSS version 11.5 (SPSS Inc., Chicago, IL). Associations between self-identified race/ethnicity and our primary outcomes were assessed using Pearson's Chi-Square and Fisher's Exact Test, depending upon the sample size for analysis, using self-identified white race as the reference group. Kappa was used to test for agreement beyond chance between adherence and perception of adherence to recommendations. All statistical tests were two-tailed.

## Results

### Respondent Characteristics

Among 393 female post-graduate physicians identified using a register of trainees, 206 were approached for participation. Response rate was > 99% (204 of 206); two declined participation, one citing urgent patient care responsibility and one citing no interest. Therefore, surveys were completed by 52% (204 of 393) of all female post-graduate physicians, representing a convenience sample.

Mean age of respondents was 30 years; 53% self-identified themselves as white, 24% as Asian, 8% as African-American, 7% as East Indian, and 5% as Hispanic/Latina (Table 1). Two elected not to identify their race/ethnicity. All were eligible for screening as none reported a hysterectomy for benign disease; 90% had at least one sexual partner in the past year, 95% in their lifetime. There were no racial/ethnic differences in age, specialty, post-graduate training year, or lifetime sexual activity (P values > 0.10).

**Table 1:** Female post-graduate resident characteristics (n = 204).^a^

**Characteristic**	
Mean age (range), y	30.1 (25 to 45)
***Self-Identified Race/Ethnicity, No. (%)***	
White	107 (53)
Asian	48 (24)
African-American	16 (8)
East Indian	15 (7)
Hispanic/Latina	11 (5)
Other	5 (3)
***Academic Department, No. (%)***	
Internal Medicine	62 (30)
Pediatrics	47 (23)
Family Medicine	15 (7)
Emergency Medicine	12 (6)
General Surgery or Surgical Subspecialty	10 (5)
OB/GYN	10 (5)
Other^b^	48 (23)
***Post-Graduate Year, No. (%)***	
One	45 (22)
Two	70 (34)
Three	54 (27)
Four or more or Fellow	35 (18)

^a ^Percentages may not sum to 100 because of rounding.

^b ^Includes 6 to 9 women from the following specialties: psychiatry, anesthesiology, neurology, radiology, and pathology.

### Racial/Ethnic Differences in Adherence

Overall, 83% of women were adherent to USPSTF screening recommendations (Table 2). Women who self-identified as Asian were significantly less adherent when compared with women who self-identified as white (69% vs. 87%; RR = 0.79, 95% CI, 0.64–0.97; P < 0.01). The rates of adherence of self-identified African-American (94%), self-identified Hispanic/Latina (91%), and self-identified East Indian women (87%) were not significantly different when compared with women who self-identified as white (87%).

**Table 2:** Proportion of female post-graduate physicians adherent to United States Preventive Services Task Force cervical cancer screening recommendations and who accurately perceived adherence or non-adherence to the recommendations, stratified by self-identified race/ethnicity

	**Adherent**		**Accurately Perceived Adherence or Non-adherence**	
	***No. (%)***	***RR (95% CI)***	***No. (%)***	***RR (95% CI)***
**Overall**	***169 (83)***	***n/a***	***167 (84)***	***n/a***
**Self-Identified Race/Ethnicity**				
**White**	93 (87)	1.00	93 (88)	1.00
**Asian**	33 (69)	0.79 (0.64–0.97)^a^	37 (82)	0.94 (0.80–1.09)
**African-American**	15 (94)	1.08 (0.93–1.25)	15 (94)	1.07 (0.92–1.24)
**East Indian**	13 (87)	1.00 (0.81–1.23)	9 (64)	0.73 (0.49–1.09)^b^
**Hispanic/Latina**	10 (91)	1.05 (0.86–1.28)	9 (82)	0.93 (0.70–1.24)

^a ^Chi-square P < 0.01, comparing women who self-identified as Asian with women who self-identified as white.

^b ^Fisher's Exact Test P = 0.036, comparing women who self-identified as East Indian with women who self-identified as white.

### Racial/Ethnic Differences in Perceptions of Adherence

Overall, 84% of women accurately perceived personal adherence or non-adherence (κ = 0.58; Table 2). Women who self-identified as East Indian were significantly less likely to accurately perceive adherence or non-adherence when compared with women who self-identified as white (64% vs. 88%; RR = 0.73, 95% CI, 0.49–1.09; P = 0.04), even though both groups of women were equally adherent (87%, as noted above). The accuracy of perceived adherence by self-identified African-American (94%), self-identified Hispanic/Latina (82%), and self-identified Asian women (82%) was not significantly different when compared with women who self-identified as white (88%).

### Racial/Ethnic Differences in Barriers to Obtaining Cervical Cancer Screening

At least one barrier to obtaining cervical cancer screening was reported by 43% of women (Table 3). Women who self-identified as Asian were significantly more likely to report any barrier to obtaining screening when compared with women who self-identified as white (60% vs. 35%; RR = 1.75, 95% CI, 1.24–2.47; P = 0.003) and there was a non-significant tendency toward women who self-identified as East Indian being more likely to report any barrier to obtaining screening when compared with women who self-identified as white (60% vs. 35%; RR = 1.74, 95% CI, 1.06–2.83; P = 0.06). The rates of reporting barriers among self-identified African-American women (25%) and self-identified Hispanic/Latina women (45%) were not significantly different when compared with women who self-identified as white (35%).

**Table 3:** Proportion of female post-graduate physicians reporting barriers to obtaining cervical cancer screening, stratified by self-identified race/ethnicity

	**Reported Barrier to Obtaining Screening,^a ^No. (%)**					
	***None***	***Any***	***"Time"***	***"MD"***	***"Comfort"***	***"Sex"***
**Overall**	***113 (57)***	***84 (43)***	***71 (36)***	***22 (11)***	***17 (9)***	***7 (4)***
**Self-Identified Race/Ethnicity**						
White	70 (65)	37 (35)	32 (30)	6 (6)	6 (6)	2 (2)
Asian	19 (40)	29 (60)	24 (50)	8 (17)	9 (19)	2 (4)
African-American	12 (75)	4 (25)	4 (25)	1 (6)	0 (0)	0 (0)
East Indian	6 (40)	9 (60)	7 (47)	5 (29)	2 (13)	3 (20)
Hispanic/Latina	6 (55)	5 (45)	4 (36)	2 (18)	0 (0)	0 (0)

Notes: "Time" = No Time to Schedule or Keep an Appointment; "MD" = No Primary Care Physician or Obstetrician-Gynecologist; "Comfort" = Not Comfortable Being Screened at Workplace Institution; "Sex" = Not Sexually Active.

^a ^Participants could report more than one barrier to obtaining cervical cancer screening.

The most frequently reported barrier was not having time to schedule or keep appointments (n = 71), which was reported by 50% of self-identified Asian women, 47% of self-identified East Indian women, and 30% of self-identified white women. Women who self-identified as Asian were more likely to report not feeling comfortable having a Pap smear at her workplace institution as a barrier to obtaining screening when compared with women who self-identified as white (19% vs. 6%; RR = 3.34, 95% CI, 1.26–8.87; P = 0.02), while women who self-identified as East Indian were more likely to report not being sexually active as a barrier to obtaining screening when compared with women who self-identified as white (20% vs. 2%; RR = 10.70, 95% CI, 1.94–58.90; P = 0.01). No women reported not having health insurance as a barrier to obtaining cervical cancer screening, nor did any women report cultural barriers to obtaining screening as an open-ended response ('cultural differences' was not explicitly provided as a multiple choice response).

## Discussion

Among a small group of female post-graduate physicians at a single institution, racial and ethnic differences in current adherence to cervical cancer screening recommendations were found. Specifically, self-identified Asian women were less adherent to recommendations, while self-identified East Indian women less accurately perceived adherence or non-adherence to recommendations, erring in their perception of being non-adherent to recommendations when they were adherent. While barriers to obtaining screening were frequently reported, self-identified Asian women were more likely to report such barriers. Self-identified East Indian women also tended to be more likely to report such barriers, although this association was not statistically significant. Moreover, across all races and ethnicities, women reported barriers that are typical of physicians in post-graduate training: no time to schedule or keep an appointment.

To our knowledge, this study is the first to examine cervical cancer screening among a racially and ethnically diverse group of post-graduate physicians, highly-educated women with professional experience in cervical cancer and prevention. Moreover, all housestaff during the time of the study were provided individual insurance policies at no cost and were able to access care either at the workplace (at on-site affiliated clinics) or outside of the workplace (at off-site affiliated or non-affiliated clinics). Because we found no differences in cervical cancer screening between self-identified white women and either African-American or Hispanic/Latina women, our study indirectly confirms prior research that demonstrated that adjusting for socioeconomic status and access to care explained observed differences in cervical cancer screening between white women and both African-American and Hispanic/Latina women.[14]

However, the observed difference in adherence to recommendations between self-identified Asian and white women, which has been shown in prior research,[15,16] suggests that culture may play a role in cervical cancer screening. This difference cannot be attributed to inaccurate perceptions of adherence to recommendations, since self-identified Asian women were as accurate as self-identified white women. Nor can this difference be attributed to limited English proficiency[15,17,18] or non-use of Western medicine,[16,25] which are unlikely to be factors among physicians training at a prestigious University hospital. There was a non-significant tendency toward self-identified Asian women being more likely to report not feeling comfortable having a Pap smear at her workplace institution, which may be a marker for differential desire for privacy or discomfort around sexuality, a predictor of cervical and breast cancer screening.[16] Prior research has suggested that post-graduate residents may not be comfortable receiving care at their institution, but no research has identified racial/ethnic differences in discomfort[26]

There are several considerations in interpreting this study. First, the survey relied on open-ended responses for report of cultural barriers to care, such as orientation towards Eastern medicine or discomfort around sexuality. Perhaps physicians in post-graduate training were preoccupied by immediate barriers to obtaining care (lack of time) and did not consider reporting cultural barriers. Second, sub-samples of post-graduate female physicians by race/ethnicity were small, prohibiting conclusive comparisons, and only half of all post-graduate female physicians at this one large academic medical center were surveyed. Third, because we initiated the study in May 2003, our opportunity to survey the substantial number of post-graduate physicians in their final year of training was small (estimated ~25% of our potential sample). However, nearly all approached post-graduate physicians completed the survey, minimizing potential selection bias, and there was no difference in survey response by academic department. Fourth, our study is based on self-reported data. The tendency of respondents to over-report health promotion and disease-prevention activities is widely recognized. [27-29] However, there is little reason to think that over-reporting would be more common among one racial/ethnic group than among another. Finally, our exploratory pilot study focused only on cervical cancer screening among post-graduate physicians at one institution and should not be generalized to utilization of other health care services. Moreover, post-graduate physicians may be particularly susceptible to poor self-care and non-utilization of recommended health care services because of the increased demands on their time and schedule. Therefore, our findings may not be representative of all practicing physicians, nor of professional women working in fields unrelated to health care such as law or business. However, this descriptive study offers an initial opportunity to examine the association of race and ethnicity and cervical cancer screening among post-graduate physicians to generate hypotheses to be tested in future research.

## Conclusion

This exploratory pilot study among insured, highly-educated physicians who have access to care found racial and ethnic differences in adherence to cervical cancer screening recommendations, suggesting that culture may play a role in cervical cancer screening. To address disparities in preventive service utilization, these results suggest that education, health literacy, insurance and access to care may be insufficient. Further work exploring cultural differences is needed.

## Declaration of Competing interests

The authors declare that they have no competing interests.

## Funding/Support

Drs. Ross and Nuñez-Smith were scholars in the Robert Wood Johnson Clinical Scholars Program at Yale University sponsored by the Robert Wood Johnson Foundation at times during their project involvement. Dr. Ross is currently supported by the Hartford Foundation and the Department of Veterans Affairs Health Services Research and Development Service project no. TRP-02-149. The views expressed in this article are those of the authors and do not necessarily represent the views of the Department of Veterans Affairs. Neither the Robert Wood Johnson nor Hartford Foundation had any role in the design or conduct of the study; collection, management, analysis or interpretation of the data; preparation, review or approval of the manuscript.

## Authors' contributions

JSR had full access to all the data in the study and takes responsibility for the integrity of the data and the accuracy of the analysis.

*Study concept and design: *JSR, BAF

*Acquisition of data: *JSR, BAF

*Analysis and interpretation of data: *JSR, MN-S, JRR

*Drafting of the manuscript: *JSR

*Critical revision of the manuscript for important intellectual content: *JSR, MN-S, BAF, JRR

*Statistical analysis: *JSR

*Administrative, technical, or material support: *JSR

*Study supervision: *JSR

## Pre-publication history

The pre-publication history for this paper can be accessed here:


